# Genomic and phenotypic evaluation of rice susceptible check TN1 collected in Taiwan

**DOI:** 10.1186/s40529-019-0269-7

**Published:** 2019-08-29

**Authors:** Yi Li, Yung-Fen Huang, Shou-Horng Huang, Yun-Hung Kuang, Chih-Wei Tung, Chung-Ta Liao, Wen-Po Chuang

**Affiliations:** 1 Department of Agronomy, National Taiwan University, Taipei, 10617 Taiwan; 2 Department of Plant Protection, Chiayi Agricultural Experiment Station, Taiwan Agricultural Research Institute, COA, Chiayi, 60044 Taiwan; 3 Crop Enviroment Division, Taichung District Agricultural Research and Extension Station, COA, Dacun Township, Changhua County, 51544 Taiwan

**Keywords:** TN1, *Cnaphalocrocis medinalis*, *Nilaparvata lugens*, Insect susceptibility

## Abstract

**Background:**

Taichung Native 1 (TN1), a variety of rice (*Oryza sativa* L.) developed in Taiwan, has played a key role in the green revolution of this major staple crop because of its semi-dwarf characteristics. Due to its susceptibility, it has been used as a susceptibility indicator in rice insect and pathogen resistance studies worldwide. While within-variety differences have been reported for agronomic traits in other rice varieties, no study has addressed the within-variety consistency of pathogen and insect susceptibility of TN1, which would influence the result interpretation of plant-pest interaction studies. Therefore, the objective of this study was to evaluate the genomic consistency and to assess a range of agronomic and insect susceptibility traits in three representative accessions of TN1 in Taiwan.

**Results:**

Among these three accessions, two were identical across 43,325 genome-wide single nucleotide polymorphisms (SNPs) while the third one differed at four SNPs. Of the three accessions of TN1, there were minor differences in seed length, seed breadth, length/width ratio, number of leaves and tillers, and number of unfilled seeds. Besides, there was no effect on relative growth rate of *Cnaphalocrocis medinalis* larvae fed on the three accession sources. Furthermore, there is no different on plant susceptibility among these three accessions against *C. medinalis* and *Nilaparvata lugens*.

**Conclusion:**

Our study indicates that it is appropriate to use TN1 in Taiwan to test for rice insect susceptibility as it yields consistent results.

## Background

Taichung Native 1 (TN1) is a variety of rice (*Oryza sativa* L.) used worldwide as a susceptible check for insect and pathogen resistance studies, such as whitebacked planthopper (*Sogatella furcifera*) (Angeles et al. 1981; Tan et al. 2004; Wu and Khush 1985; Yang et al. 2014), brown planthopper (*Nilaparvata lugens*) (Deen et al. 2017; Du et al. 2009; Jing et al. 2014; Tan et al. 2004), bacterial blight (*Xanthomonas oryzae*) (Kumar et al. 2012; Lee et al. 2003; Tseng et al. 2015; Yugander et al. 2018), Asian rice gall midge (*Orseolia oryzae*) (Rawat et al. 2013), green leafhopper (*Nephotettix virescens*) (Vu et al. 2014), rice leaffolder (*Cnaphalocrocis medinalis*) (Guo et al. 2019; Han et al. 2015), rice stem borer (*Chilo suppressalis*) (Liu et al. 2018). Developed in Taiwan in the 1950s, TN1 was the first semi-dwarf rice variety in the world created by intended hybridization (Huang et al. 1972), and it played a key role in the green revolution of rice. TN1 was widely adopted by farmers within Taiwan soon after its release, because of its high yielding potential (Huang et al. 1972), and the area under cultivation reached more than one million hectares in India by 1967 (Chandler 1992). However, the planting area of TN1 subsequently declined quickly due to its poor grain quality and susceptibility to disease and insects (Huang et al. 1972). These features transformed it into a standard susceptibility check variety for disease and insect resistance studies worldwide.

The host-plant resistance (HPR) strategy was first defined by Reginald Painter in 1951(Painter 1951) as the effects of interactions between plant and insect. Painter defined three plant resistance categories, such as antibiosis, antixenosis, and tolerance (Painter 1951). Antibiosis would cause detrimental effects on insect survival, whereas antixenosis would affect on insect behavior. Plants have ability to repair or recover from damage would have tolerance ability. Ever since, studies on insect-resistance genes have been developed, although HPR is now extended to various environmental stress, including abiotic and biotic stresses. In rice, TN1 has been used as a susceptible variety for resistance against the brown planthopper (*N. lugens*) since 1969 (Pathak et al. 1969) as well susceptible check for pathogen management since 1980 (Shukla and Anjaneyulu 1980).

Worldwide, TN1 continues to be routinely used in insect and pathogen resistance studies (Deen et al. 2017; Yugander et al. 2018), where each research laboratory maintains its own TN1 seed stock. Although rice is self-fertilizing, occasional pollen transfer occurs due to environmental conditions and interactions with the local insect fauna; this may lead to some local genetic variations within a variety. The Japanese rice variety “Koshihikari” has been cultivated in Taiwan since 1977 and has subsequently developed three ecotypes based on agronomy trait analysis (Lin and Cheng 2012). The latter study indicates that a rice variety may vary from its original line with time. Such variations in TN1 may affect its response to insect and pathogen resistance in susceptibility tests. However, the uniformity of the extent of susceptibility across different stocks of TN1 since its spread in the 1950s has not been addressed. Therefore, it is essential that susceptibility checks for insect and pathogen resistance in varieties such as TN1 are stable and consistent to allow robust comparisons of data derived from different laboratories. However, no data is currently available in terms of consistency of insect and disease susceptibility to different sources of TN1. Given (i) the importance of consistent susceptibility expression in varieties undergoing pest resistance studies (ii) the popularity of TN1 as a variety to check susceptibility, and (iii) the lack of information on the stability of susceptible phenotypes across different accessions in TN1, this study addressed the genomic and phenotypic variation of a set of TN1 accessions that are currently representative of TN1 sources native to Taiwan.

## Materials and methods

### Plant materials

**Fig. 1:**
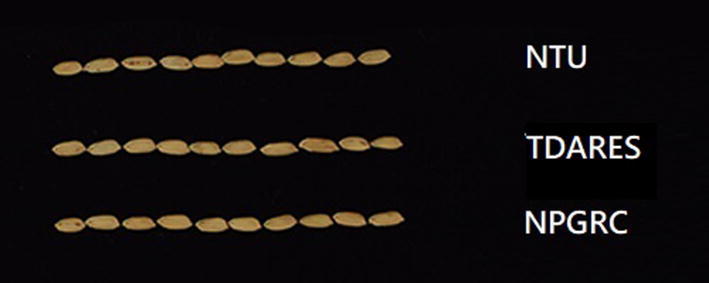
Grains of the three accessions of the Taichung Native 1 (TN1) rice variety

TN1 seed was obtained from the National Plant Genetic Resources Center (NPGRC), Taichung District Agricultural Research and Extension Station, Council of Agriculture, Changhua (TDARES, where TN1 was bred), and National Taiwan University (NTU; Fig. 1). Seeds of a *C. medinalis*-resistant rice variety, Qingliu (Liao and Chen 2017), were obtained from TDARES. Qingliu plants have higher mortality rate and lower growth rate on *C. medinalis* larvae than TN1 plants (Guo et al. 2019).

### Genotyping and genotypic data analysis

DNA extraction and genotyping-by-sequencing (GBS) library preparation of rice accessions were performed following the method reported by Chang et al. (2017) and Elshire et al. (2011), where 100 ng of genomic DNA from each accession was digested using ApeKI and ligated with specific barcoded-adaptor for PCR amplification. Single-end reads (ca. 125 bp) in FASTQ format were then processed using the GBS pipeline with default parameters in the TASSEL 3 software on Linux operating system, Os-Nipponbare-Reference-IRGSP-1.0 (IRGSP-1.0) was served as reference sequence for short reads alignment. After removing erroneous genotypic scores, we replaced missing data using major alleles within each accession. Only SNPs without missing data across the four accessions were used for further comparison. For each SNP, genotypic scores were converted to the number of major alleles (e.g., 0, 1, and 2), and Euclidean distance was calculated for each pair of individuals using the “dist” function implemented in R 3.5.0 (R Core Team 2018). The complete linkage method was used for hierarchical clustering based on Euclidean distance using “hclust” implemented in R.

### Agronomic characterization of TN1

Seeds were first sterilized using 2% (v/v) NaOCl for 30 min, and then washed with sterile water. The sterilized seeds were placed on water-moistened filter paper in Petri dishes and incubated under dark conditions at 37 °C for 48 h. Germinated seeds of similar size were selected for planting in a peatmoss mix (Da Chiang Chun Horticulture Material Co. LTD., Nantou County, Taiwan) and, at the two-leaf stage, 10 replicates of two seedlings of each variety were transferred into plastic pots filled with soil (243 × 243 × 257 mm; Keyway Co., Taoyuan, Taiwan). Plants were put based on completed randomized design and were maintained in a glasshouse at the National Taiwan University from July 9 to December 2, 2016. Agronomic traits were recorded from 10 plants per variety, where plant height was taken at harvest and measured as the distance from the soil surface to the tip of the main panicle (cm); leaf stage was recorded from germination until harvest; numbers of mature and immature panicles were counted manually. When harvested, seeds were dried at 60 °C for 10 days. The number of filled seeds and unfilled seeds were counted using a Numigral seed counter (Tripette et Renaud, Paris, France). Three mid-sized seeds selected from five seeds per plant with ten plants gave a total of thirty seeds for each TN1 accession; from these, seed dimensions (mm) were recorded using Vernier calipers (Central Scientific Company, Chicago, U.S.A.), and seed length/width ratio was calculated by diving length by width.

### Insect rearing

A *C. medinalis* colony, which had been collected from a rice field near Taichung, Taiwan, was obtained from TDARES, and was reared on corn seedlings (“White pearl”, Known-You Seed Co., Taiwan) in insect cages (BugDorm-4, MegaView, Taiwan) following the modified corn seedling rearing method described by Shono and Hirano (1989). Larvae at the V3 growth stage were transferred onto corn seedlings planted in vermiculture, and the emerged adults were fed a 10% (v/v) sucrose solution. Newly hatched neonates and third instar larvae from the emerged adults were used in this study. They were reared in growth chambers set to a 12 h:12 h (day/night) photoperiod at 30/25 °C and 55 ± 5% relative humidity.

A *N. lugens* colony was reared on TN1 plants in wire mesh cages (L × W × H: 50 by 50 by 90 cm) with greenhouse conditions (temperature ranged from 25 to 35 °C with 12 h:12 h (day/night) photoperiod).

### Relative growth rate of *C. medinalis* larvae

Third instar *C. medinalis* larvae were starved for 2 h and then placed on the youngest fully developed leaves of 30-days old TN1 plants (one larva per plant) in a mesh-covered cage for 6 days. Relative growth rate was calculated as [(final weight of insect − initial weight of insect)/average weight between final and initial weight of insect)]/duration (days) (Farrar et al. 1989; Waldbauer 1968).

### *C. medinalis* susceptibility

Neonates were placed on the newly expanded leaves of 10 plants (three larvae per plant) at the maximum tillering stage, and the number of damaged and rolled leaves were recorded after 7 and 15 days.

### *N. lugens* susceptibility

Standard seedbox screening test (SSST) was used to evaluate the TN1 susceptibility against *N. lugens* (Velusamy et al. 1986). Briefly, twenty-four seeds of each accession were sown in lines. Only twenty seedlings from each accession were selected to perform the SSST. At 14 days after sowing, seedlings were infested with 2nd to 3rd instar *N. lugens* nymphs at a density of 10 nymphs per seedling. The damage rating was using the standard evaluation system in rice for *N. lugens* infestation where 0 = no injury, 1 = slightly damage, 3 = 1st and 2nd leaves of plants partially yellowing, 5 = 10% to 25% plants with pronounced yellowing and stunting or wilting symptoms, 7 = more than half of plants wilted, 9 = all plants wilted or dead (IRRI 2002). This SSST experiment was repeated three times for the analysis.

### Statistical analysis

Genotype-source differences in seed characteristics, agronomic traits, number of rolled leaves, *C. medinalis* relative growth rates, and *N. lugens* susceptibility were tested using analysis of variance (ANOVA); least significant difference (LSD) was used to test for differences within the genotype-sources at P < 0.05. Data were analyzed using the free statistical software platform, R (version 3.5.0) (Team 2018).

## Results

### Genotype-source genomics

We identified 44,142 SNPs across four rice accessions: Qingliu, TN1-NPGRC, TN1-TDARES, and TN1-NTU were sequenced four, six, five, and six times, respectively. One individual of Qingliu showed particularly high heterozygosity (11.6%) compared with the other genotypes (< 0.05%). Heterozygosity in selfing plants, such as rice, tends to indicate genotyping errors during sample preparation or sequencing, therefore we removed this Qingliu individual; this left three remaining individuals. Before the genotypic scores of the available individuals were combined to create a representative genotype for each accession, 7744, 4543, 7843, and 11,990 SNPs had at least one missing data point for Qingliu, TN1-NPGRC, TN1-TDARES, and TN1-NTU, respectively. After data imputation using major alleles, the number of SNPs with missing scores reduced to 655, 183, 206, and 151, respectively. As a result, we used a final dataset of 43,325 SNPs without missing data to compare the four genotype-source combinations (Additional file 1: Table S1).

**Fig. 2:**
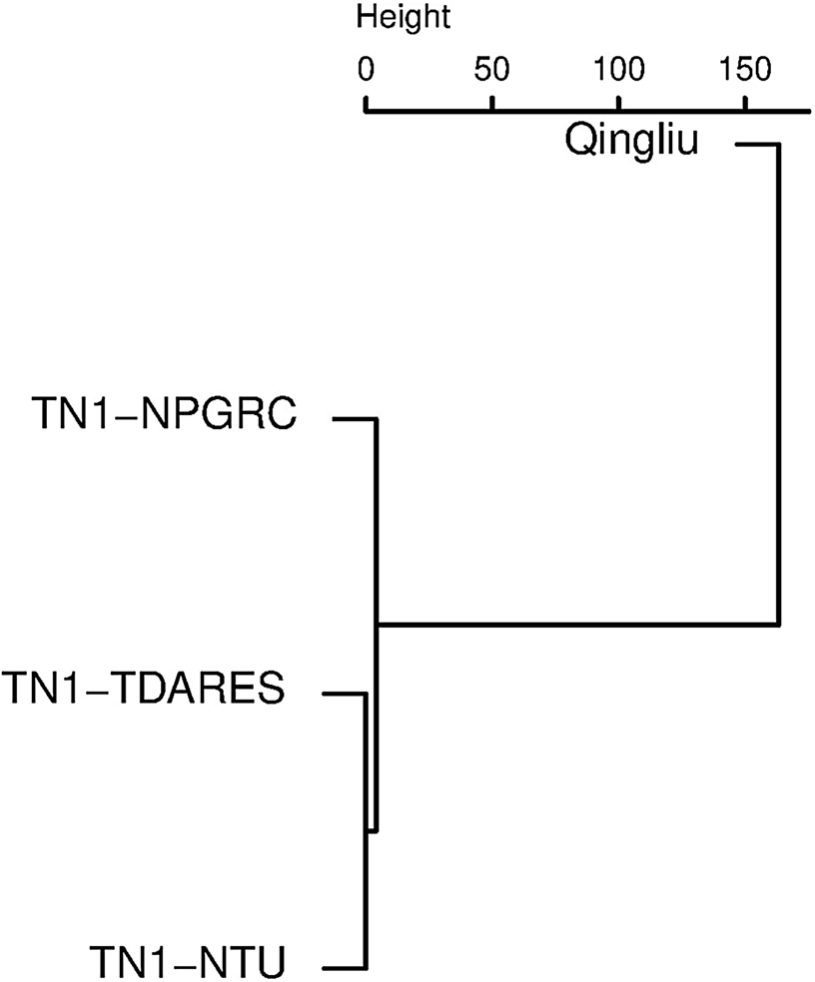
Hierarchical clustering of Qingliu and three accessions of the Taichung Native 1 (TN1) rice variety based on Euclidian distance calculated from 43,325 genome-wide SNPs

Among the TN1 accessions, TN1-TDARES and TN1-NTU were found to be 100% identical, while TN1-NPGRC differed from both accessions at four SNPs, and Qingliu differed from TN1-TDARES and TN1-/NTU at 6659 SNPs (Fig. 2). The four polymorphic SNPs within TN1 accessions were S2_9566762 (G/A), S7_27071173 (A/G), S10_8786836 (A/G), S12_25809234 (T/C), where the markers were named after its physical position on the reference genome IRGSP-1.0 (chromosome_position). The three former SNP were located in intergenic regions or intron of a predicted gene without transcript evidence (S2_9566762) while S12_25809234 would cause a change in amino acid (Aspartic acid–Asparagine) of an uncharacterized protein (locus *Os12g0610800*). The difference in genomic distribution of SNPs between Qingliu and TN1 showed no special clustering and was quite even (Additional file 2: Figure S1).

### Seed characteristics, agronomic traits

TN1 seeds from TDARES were longer and broader, with a greater length/width ratio than the other accessions, and TN1 seed dimensions from NTU and NPGRC were similar (P < 0.05), but seed width did not vary among different accessions (P = 0.579 and P = 0.782, respectively; Table 1). TN1-NTU had fewer leaves than TDARES and NPGRC and more tillers than NPGRC (P < 0.05). There were no differences in the number of mature and immature panicles and number of filled seeds among the three accessions of TN1 (P = 0.100 and 0.917, respectively; Table 1). However, TN1-NTU had more unfilled seeds than the other two accessions (P = 0.02, Table 1).

**Table 1:** Seed characteristics and agronomic traits of three accessions of the Taichung Native 1 (TN1) rice variety

Variable	NTU	TDARES	NPGRC
Seed characteristics			
Length (mm)	69.6 ± 0.4 b	71.9 ± 1.1 a	68.8 ± 0.5 b
Width (mm)	26.0 ± 0.3	25.6 ± 0.4	25.5 ± 0.3
Breadth (mm)	18.0 ± 0.1 a	17.8 ± 0.1 a	17.1 ± 0.2 b
Length/width ratio	2.7 ± 0.0 b	2.8 ± 0.1 a	2.7 ± 0.0 b
Agronomic traits			
Plant height (cm)	114.20 ± 2.1	115.28 ± 1.7	116.71 ± 3.4
No. of leaves	14.0 ± 0.2 b	14.8 ± 0.2 a	15.0 ± 0.3 a
No. of tillers	11.6 ± 0.4 a	9.9 ± 0.9 ab	8.1 ± 0.5 b
No. of panicles			
Mature	10.8 ± 0.7	9.2 ± 0.9	8.8 ± 0.5
Immature	3.0 ± 0.9	2.5 ± 0.9	2.7 ± 0.8
No. of seeds			
Filled	515.7 ± 18.1	553.7 ± 45.2	548.3 ± 34.3
Unfilled	329.2 ± 28.2 a	213.3 ± 23.2 b	234.0 ± 34.3 b

Different letters indicate differences between TN1 genotype sources

### Relative growth rate of *C. medinalis*

The relative growth rate of *C. medinalis* larvae fed on three TN1 accessions ranged from 0.193 to 0.206; the findings were not significant (P = 0.1454; Table 2).

**Table 2:** Relative growth rates of *Cnaphalocrocis medinalis* larvae fed on three accessions of the Taichung Native 1 (TN1) rice variety

	NTU	TDARES	NPGRC
Relative growth rate	0.206 ± 0.004	0.193 ± 0.007	0.206 ± 0.005

### TN1 susceptibility against *C. medinalis* and *N. lugens*

The number of rolled leaves ranged between 12.6 to 13.0 among the three TN1 accessions when infested with *C. medinalis*, but there was no significance (P = 0.9885; Table 3). The injury rating of three TN1 accessions against *N. lugens* ranged from 7.67 to 8.33; the findings were not significant (P = 0.729; Table 4).

**Table 3:** Effect of *Cnaphalocrocis medinalis* infestation on leaf rolling in three accessions of the Taichung Native 1 (TN1) rice variety

	NTU	TDARES	NPGRC
*C. medinalis* infestation			
No. of rolling leaves	12.6 ± 2.0	12.8 ± 2.8	13.0 ± 1.1

**Table 4:** Injury rating of three accessions of the Taichung Native 1 (TN1) rice variety infested by *N. lugens*

	NTU	TDARES	NPGRC
Damage rate	8.33 ± 0.67	7.67 ± 0.67	8.33 ± 0.67

## Discussion

Since TN1 was developed in Taiwan, we collected three representative accessions (NPGRC, TDARES, and NTU) of TN1 in Taiwan to investigate whether genomic and phenotypic within-variety variation exist for TN1. The high-throughput SNP data showed that the three TN1 accessions were almost genetically identical (Fig. 2). Little phenotypic difference was observed in terms of seed characteristics and agronomic traits, and the sole difference in seed length, breadth, length/width ratio were minor. TN1-NTU tends to have fewer leaves and more tillers compared with the other two accessions (Table 1). We hypothesized that the higher number of unfilled seeds in TN1-NTU was due to the presence of more ineffective tillers.

Besides genotypic data and agronomic traits, susceptibility to two major rice pests (*C. medinalis* and *N. lugens*) in Asia were investigated in this study. These two insect species represent not only important rice pests but also two different feeding styles (chewing and piercing-sucking). Plants may be attacked by various species of phytophagous insects with different feeding guilds. Some insects feed on plants by chewing or tearing tissues, while other insects feed on plants by inserting needle-like mouthparts (stylets) into plant cells (Bonaventure 2012). The defensive mechanisms of plants response to different insect feeding styles are usually distinct. When insects feed on plants, components in insect saliva or regurgitant would have chance to get into plant cells and further trigger the plant defense, called herbivore-associated molecular patterns (HAMPs) or damage associated molecular patterns (DAMPS) (Nguyen et al. 2016). For example, feeding of *Manduca sexta* larvae induces the accumulation of jasmonic acid (JA) and ethylene (ET) in *Nicotiana attenuata*, whereas the phloem-sap insect *N. lugens* induces salicylic acid (SA) pathway in rice (Diezel et al. 2009; Guo et al. 2014). Furthermore, induced JA and SA were observed in infested rice plant upon *C. medinalis* infestation (Ye et al. 2012). Thus, it is important to evaluate not only the susceptibility but also the response stability among these three TN1 accessions. In this study, two measurements of plant response to *C. medinalis* and *N. lugens* were used to represent the phenotyping results. The *C. medinalis* and *N. lugens* susceptibility of TN1 represented the plants response against insects. In addition, the relative growth rate of *C. medinalis* fed on TN1 represented insect performance on this diet. Since TN1 is used to check insect and pathogen resistance, plant response and insect performance are the two indicators of TN1 responses. Based on our results (Tables 2, 3, 4), these two measurements confirmed that both plant response and insect performance were consistent between these three accessions. This study confirmed that TN1 accessions from Taiwan are appropriate to be used as susceptibility checks as their testing yielded consistent results.

This is the first study to use both genomic and phenotypic data to re-evaluate and to characterize the susceptibility of “TN1”, a widely-used check variety in insect and pathogen resistance studies. Although only three accessions were investigated in this study, they were representative of the available TN1 sources in Taiwan. The National Plant Genetic Resources Center in Taiwan is the government-owned seed bank that provides germplasms for scientific research, both in Taiwan and worldwide. Taichung District Agricultural Research and Extension Station is the original place where TN1 was bred. The third accession of TN1 was obtained from stock belonging to the university, which had been increased through several generations. Across 43,325 genome-wide SNPs, the accession from NTU and the one from TDARES showed 100% identity while the accession from NPGRC differed at only four SNPs from them and the four SNPs were located either within intergenic region or intron. The only SNP causing an amino acid change (S12_25809234) located in a predicted gene coding for an uncharacterized transmembrane protein and the corresponding amino acids (Asp–Asn) differ only by a terminal amide group in the side chain which can be spontaneously lost from proteins by deamination. Therefore, these four SNPs do not seem to cause critical difference between accessions. Environmental change and frequency of insect occurrence can affect local adaptation. Thus, we were eager to determine the variation among these three accessions on the island before re-evaluating TN1 accessions worldwide. This study should assist rice breeders around the world to re-evaluate their own TN1 lines. If breeders have concern regarding their own accessions, we highly recommend that they request a TN1 accession from NPGRC or TDARES in Taiwan.

## Conclusions

The genomic and phenotypic data in this study demonstrates that three representative TN1 accessions in Taiwan were consistent. These three accessions could be used for the worldwide standard of TN1.

## Supplementary information

**Additional file 1: Table S1.** SNPs across four rice accessions: Qingliu, TN1-NPGRC, TN1-TDARES, and TN1-NTU.

**Additional file 2: Figure S1.** Genomic distribution of 43,325 SNPs used in this study. Available SNPs are indicated in gray or blue, where blue indicates polymorphic SNPs between Qingliu and TN1. Position of centromere is indicated by a black diamond.

## Data Availability

The data used and analyzed for the current study can be obtained from the corresponding author.
