# Supplementary figures and images for: Genomic and phenotypic evaluation of rice susceptible check TN1 collected in Taiwan
Source: Bot Stud. 2019 Aug 29;60:19. doi: 10.1186/s40529-019-0269-7 (PMC6715756; doi:10.1186/s40529-019-0269-7)

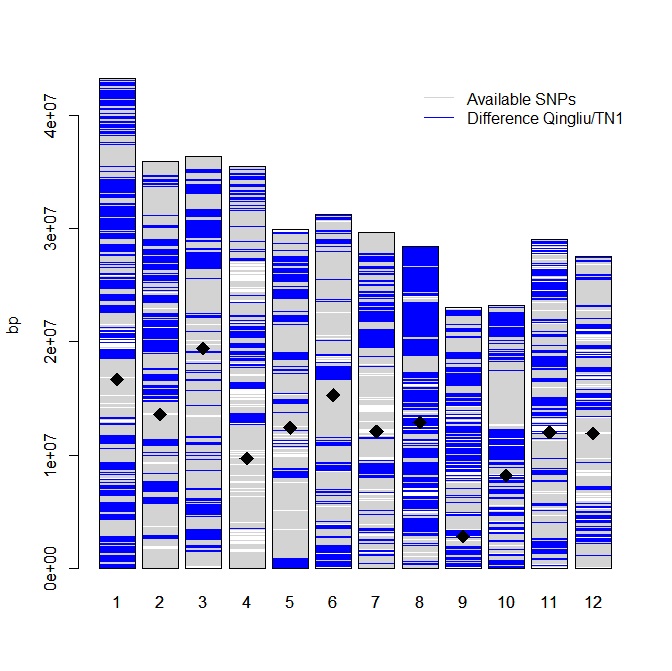

Supplement: Supplementary file 2 — Additional file 2: Figure S1. Genomic distribution of 43,325 SNPs used in this study. Available SNPs are indicated in gray or blue, where blue indicates polymorphic SNPs between Qingliu and TN1. Position of centromere is indicated by a black diamond. [file 40529_2019_269_MOESM2_ESM.jpg]
